# Prevalence and risk factors for soil-transmitted helminth infection in mothers and their infants in Butajira, Ethiopia: a population based study

**DOI:** 10.1186/1471-2458-10-21

**Published:** 2010-01-19

**Authors:** Yeshambel Belyhun, Girmay Medhin, Alemayehu Amberbir, Berhanu Erko, Charlotte Hanlon, Atalay Alem, Andrea Venn, John Britton, Gail Davey

**Affiliations:** 1 Aklilu Lemma Institute of Pathobiology, Addis Ababa University, Addis Ababa, Ethiopia; 2 School of Public Health, Addis Ababa University, Addis Ababa, Ethiopia; 3 Division of Epidemiology & Public Health, the University of Nottingham, Nottingham, UK; 4 Department of Psychiatry, Addis Ababa University, Addis Ababa, Ethiopia

## Abstract

**Background:**

Soil-transmitted helminths (STHs) are widespread in underdeveloped countries. In Ethiopia, the prevalence and distribution of helminth infection varies by place and with age. We therefore investigated the prevalence of and risk factors for STH infection in mothers and their one year-old children living in Butajira town and surrounding rural areas in southern Ethiopia.

**Methods:**

In 2005-2006, 1065 pregnant women were recruited in their third trimester of pregnancy. In 2006-2007, when children reached their first birthdays, data on the infants and their mothers were collected, including stool samples for qualitative STH analysis. Questionnaire data on various demographic, housing and lifestyle variables were available. Logistic regression analysis was employed to determine the independent risk factors for STH infection in the mothers and children.

**Results:**

908 mothers and 905 infants provided complete data for analysis. Prevalence of any STH infection was 43.5% (95% confidence interval (CI) 40.2-46.8%) in mothers and 4.9% (95%CI 3.6-6.5%) in children. In the fully adjusted regression model, infrequent use of soap by the mother was associated with increased risk (odds ratio (OR) 1.40, 95% CI 1.04-1.88, and 1.66, 95% CI 0.92-2.99, for use at least once a week and less frequent than once a week respectively, relative to daily use; p for trend = 0.018), and urban place of residence (OR 0.45, 95% CI 0.28-0.73, p = 0.001) was associated with reduced risk of maternal STH infection. The only factor associated with STH infection in infants was household source of water, with the greatest risk in those using piped water inside the compound (OR 0.09, 95% CI 0.02-0.38 for river water, 0.20, 95% CI 0.56-0.69 for either well or stream water and 0.21, 95% CI 0.09-0.51 for piped water outside compared with piped water inside the compound, overall p = 0.002)

**Conclusion:**

In this rural Ethiopian community with a relatively high prevalence of STH infection, we found a reduced risk of infection in relation to maternal hygiene and urban living. Daily use of soap and a safe supply of water are likely to reduce the risk of STH infection.

## Background

Soil-transmitted helminths (STHs) are a group of nematodes that infect more than a billion people worldwide [1]. Of particular public health importance are *Ascaris lumbricoides, Trichuris trichiura *and hookworms [1,2]. *Strongyloides stercoralis *is also a STH [3], although detailed information on the prevalence is lacking. In Ethiopia the prevalence of hookworm is estimated to be 16% and the prevalences of *A. lumbricoides *and *T. trichiura *are 37% and 30% respectively [4]. STH infections have not been targeted for control in Ethiopia [4], though mass de-worming as a component of the Enhanced Outreach Strategy (EOS) targeting under five children started in 2004 [5].

In developing countries, where control measures are often difficult to implement, STHs remain a significant health problem [6,7]. It is estimated that between a quarter and a third of pregnant women in sub-Saharan Africa are infected with hookworm and are at risk of preventable hookworm-related anemia [8]. Many children living in endemic areas are infected continuously from soon after birth and often experience chronic ill health [1,9]. Infections with STHs may have important health consequences during this developmental stage, affecting child health and development, slowing down growth and reducing adults productivity and work capacity [1,6,7,9]. These helminth infections may also elicit potent immune responses through effects on the immune system [1,10].

Intervention against STH infection is based on regular anti-helminthic treatment, improved water supply, sanitation and health education [11]. Low-cost, high-coverage delivery of anti-helminthic treatment has been achieved in some settings [12], but improving sanitation is more complex. In Ethiopia, for example, levels of access to improved sanitation in rural areas are very low (5.4%) [13], making evaluation of other components of intervention important. In this study, we present data from an area in which intermittent mass de-worming has been carried out [14], but where levels of sanitation are still rudimentary. We present data on prevalence of STH infection among mothers and one-year old children in a rural area of Ethiopia using a population-based birth cohort to define the prevalence of STH infection and identify factors amenable to intervention.

## Methods

### Study area and population

The study site was the Butajira area, which is located in Gurage zone, Southern Ethiopia. The capital Butajira lies at a distance of 130 Km from the capital, Addis Ababa. The area is predominately rural and most residents live in villages as agriculturists growing coffee, maize, *teff (Eragrostos tef)*, pepper, the stimulant *khat *(*Catha edulis forskal*) and *enset *(false banana) [14]. The total population of the study area is approximately 250,000, with a diverse multi-ethnic group; the *Gurages *(Meskan, Mareko and Silti) form the majority [14]. Deworming started in the study area in 2006 as part of the Enhanced Outreach Strategy (EOS) [15]. Antihelminthics have been given regularly in ten successive rounds in under five children, covering 41 administrative units of Butajira district and its surroundings [15].

### Study design and procedures

**Figure 1:**
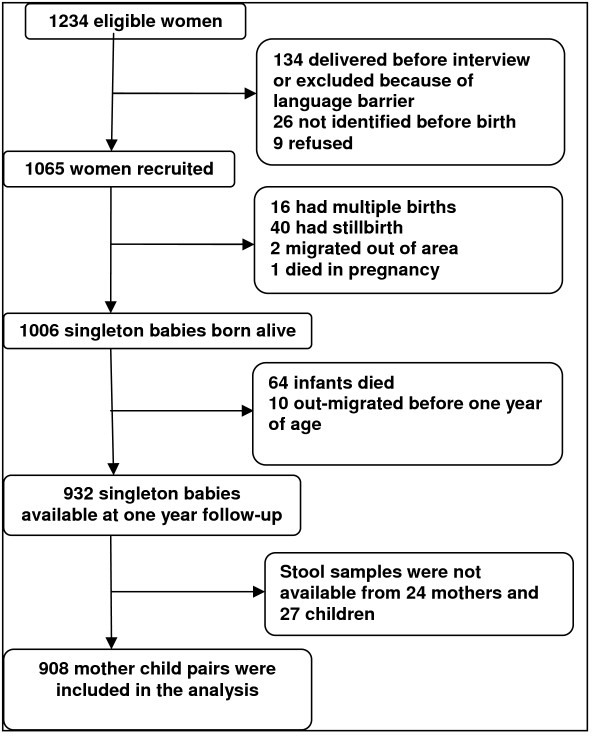
**Study cohort**.

The Butajira Rural Health Project (BRHP), a Demographic Surveillance Site (DSS) covering nine rural and one urban administrative area, was established in 1987 [14]. The DSS population currently is around 60,000 with more than 13,000 women of reproductive age. Between July 2005 and February 2006 all pregnant women in their third trimester and aged 15-49 years were identified from the BRHP and a birth cohort established, details of which have been reported elsewhere [16]. Of the 1234 eligible women identified, 1065 were recruited and these women gave birth to 1006 live singleton babies (Fig 1). Those mother-child pairs still living in the area and alive (n = 932) were followed up and data were collected between July 2006 and June 2007, at the time of the child's first birthday.

### Data collection

After written consent was obtained from the mothers of participating children, stool samples were collected from both the mother and child. Samples were collected by data collectors known to the mothers and children since the initiation of the birth cohort. The stool samples were placed in labeled plastic tubes containing 10% formalin and transported for analysis to the Aklilu Lemma Institute of Pathobiology, Addis Ababa University. Samples were then examined qualitatively using the formol-ether concentration method [17]. Information on demographic and lifestyle factors was collected by interviewer-administered questionnaire during initial recruitment of the mother during pregnancy and at the first year follow up.

### Data management and analysis

Data on parasites were entered into Excel and manually checked for error against the hard copies. Questionnaire data were double-entered into EpiData version 3. Analysis was performed using SPSS version 12.0.1 (SPSS Inc. Chicago, IL). The dependent variables were any STH infection in the mother and any STH infection in the child (where STH infection is taken to mean infection with any of *A. lumbricoides, T. trichiura*, hookworm or *S. stercoralis*). Potential risk factors explored were demographic factors (gender, mother's age, urban/rural place of residence and ethnicity), markers of social advantage (education, occupation, home ownership and TV/radio ownership) and factors associated with housing and lifestyle (roof type, type of toilet, water source, animals in the home, place of cooking, soap use, and waste disposal method). Initially the univariate association between each exposure and the presence of infection was assessed using the Chi-squared test and odds ratios were computed as measures of association. To determine the independent risk factors for infection, logistic regression analysis was employed. Variables were manually added and removed from the model until only those that significantly improved the model remained. Urban/rural place of residence and gender (child model only) and additionally age of the mother (maternal model only) were treated as *a priori *confounders and retained in the model regardless of significance.

### Ethical consideration

Approval for this study was granted by the Ethics Committee of the Aklilu Lemma Institute of Pathobiology, Addis Ababa University. All mothers and children with STH infections were treated with anti-helminthics based on the national protocol.

## Results

### Description of the study subjects

Nine hundred thirty two child-mother pairs were followed up at one year, and 908 (97.4%) of the mothers and 905 (97.1%) of their infants provided adequate stool samples and were included in the analysis (Fig 1). The majority were from rural areas (87%) and there were roughly equal numbers of male (460; 50.7%) and female (448; 49.3%) infants. The age range of interviewed mothers was 15 to 44 years (mean, standard deviation [SD] 26.8 [6.3] years), with almost one third (38.7%) aged under 25.

### Prevalence of soil-transmitted helminths

Four species of soil-transmitted helminths (STHs) were identified in the stool samples, with the overall prevalence of any STH infection being 43.5% (95% confidence interval (CI) 40.2-46.8%) in mothers, and 4.9% (95%CI 3.6-6.5%) in children, respectively (Table 1). Hookworm was the predominant intestinal helminth infection, detected in 36.1% of mothers and in 2.3% of children, and *A. lumbricoides *was the second most frequently detected intestinal parasite with prevalence of 8.8% in mothers and 1.5% in children (Table 1). About one third (36.2%) of mothers and 4.4% of children had a single infection, while 6.6% of mothers and 0.4% of children had double infections, 0.7% of mothers had triple infections (Table 1).

**Table 1:** Prevalence of soil-transmitted helminth infections in mothers and their infants in the Butajira birth cohort, Ethiopia, 2007

*Soil-transmitted helminths*	Children (N = 905) No (%)	Mothers (N = 908) No (%)
*Hookworms*	21 (2.3)	328 (36.1)
*Ascaris lumbricoides*	13 (1.5)	80 (8.8)
*Trichuris trichiura*	4 (0.4)	30 (3.3)
*Strongyloides stercoralis*	10 (1.1)	29 (3.2)
***Any soil-transmitted helminth infection***	**44 (4.9)**	**395 (43.5)**
*Prevalence of single infection*	40 (4.4)	329 (36.2)
*Prevalence of double infection*	4 (0.4)	60 (6.6)
*Prevalence of triple infection*	0 (0)	6 (0.7)

N = total number of study participants

### Univariate risk factor analysis for STH infection in mothers

The distributions of the demographic and lifestyle factors of the subjects are shown in Tables 2 and 3. In univariate analyses, significant positive associations were seen between presence of any STH in the mother and living in a house with a thatched roof (Odds Ratio (OR) 1.62; 95% CI 1.18-2.24, p = 0.003), home ownership (OR 1.67, 95% CI 1.04-2.68, p = 0.033), cooking inside (OR 1.68; 95% CI 1.18-2.37, p = 0.003) and for disposing waste in open field relative to burying or burning (overall p = 0.013, OR 1.66; 95% CI 1.23-2.46). In addition, frequency of soap use was significantly associated with maternal infection with increased risk seen for infrequent soap users compared to daily use (overall p = 0.008, OR 1.51, 95% CI 1.14-2.01). A significant negative association was found in relation to urban living (OR 0.48; 95% CI 0.32-0.73, p = 0.001), whereas the association with education level was only of borderline significance (p for trend = 0.051 OR 0.71; 95% CI 0.51-1.00) (Tables 2 and 3).

**Table 2:** Univariate analysis of socio-demographic characteristics and their association with soil-transmitted helminths in the birth cohort, Butajira Ethiopia, 2007

Demographic variables	N (%)	Soil-transmitted helminths in the mother (N = 908)			Soil-transmitted helminths in the child (N = 905)		
		
		*n (%)*	*Crude OR (95%CI)*	*P-value*	*n (%)*	*Crude OR (95%CI)*	*P-value*
*Male gender*	460 (50.7)	-	-	-	18 (3.9)	0.66 (0.36, 1.23)	0.190
*Urban residence*	118 (13.0)	37 (31.4)	0.48 (0.32,0.73)	0.001	10 (8.6)	2.07 (1.00, 4.32)	0.052
*Mother's Age*				0.949			0.442
15-24	351 (38.7)	155 (44.2)	1	0.759^$^	21 (6.0)	1	0.224^$^
25-34	424 (46.7)	183 (43.2)	0.96 (0.72,1.28)		18 (4.3)	0.70 (0.37, 1.33)	
35-44	133 (14.7)	57 (42.9)	0.95 (0.63,1.42)		5 (3.8)	0.61 (0.23, 1.66)	
*Ethnicity*				0.280			0.550
Meskan	429 (47.3)	183 (42.7)	1		21 (4.9)	1	
Mareko	118 (13.0)	57 (48.3)	1.26 (0.83,1.89)		8 (6.8)	1.41 (0.61, 3.27)	
Silti	211 (23.2)	98 (46.5)	1.17 (0.84,1.62)		7 (3.3)	0.67 (0.28, 1.60)	
Others*	150 (16.5)	57 (38.0)	0.82 (0.56,1.21)		8 (5.4)	1.10 (0.48, 2.54)	
*Mother's Education*				0.138			0.124
No education	639 (70.2)	290 (45.4)	1	0.051^$^	32 (5.0)	1	0.830^$^
Read write^£^	86 (9.5)	37 (43.0)	0.91 (0.58, 1.43)		1 (1.2)	0.22 (0.03, 1.65)	
Formal education	183 (20.2)	68 (37.2)	0.71 (0.51, 1.00)		11 (6.0)	1.22 (0.60, 2.46)	
*Mother's Occupation*				0.428			0.700
House wife	762 (83.9)	338 (44.4)	1		38(5.0)	1	
Trade related	104 (11.5)	42 (40.4)	0.85 (0.56,1.29)		5 (4.9)	0.97 (0.37,2.52)	
Others^♀^	42 (4.6)	15 (35.7)	0.70 (0.36, 1.33)		1 (2.4)	0.46 (0.06, 3.46)	
*Home ownership*	822 (90.5)	367 (44.7)	1.67 (1.04, 2.68)	0.033	37 (4.5)	0.53 (0.23, 1.24)	0.143
*TV/Radio ownership*	466 (51.4)	197 (42.3)	0.91 (0.70, 1.18)	0.467	27 (5.8)	1.52 (0.82, 2.84)	0.184

* Sodo, Dobi, Oromo, Amhara,

^♀ ^Farm related, profession related, trade related and daily laborer, ^$^p for trend

^£ ^read and write but have no formal education

N = total number of study participants, n = number of outcome for each exposure

**Table 3:** Univariate analysis of potential risk factors of soil-transmitted helminths in mother and their infants in the Butajira birth cohort, Ethiopia, 2007

Risk factor		Soil-transmitted helminths in the mother (N = 908)			Soil-transmitted helminths in the child (N = 905)		
		
	N(%)	*n(%)*	*Crude OR (95%CI)*	*P-value*	*n(%)*	*Crude OR (95%CI)*	*P-value*
*Living in thatched house vs. corrugated sheet*	697 (76.8)	322 (46.2)	1.62 (1.18,2.24)	0.003	28 (4.0)	0.51 (0.27,0.96)	0.037
*Toilet use vs. open field§*	564 (62.1)	234 (41.5)	0.81 (0.62,1.06)	0.080	32 (5.7)	1.66 (0.84,3.26)	0.144
*Main water source*				0.188			<0.001
Inside pipe^§^	54 (6.0)	17 (31.5)	1		10 (18.5)	1	
Outside pipe^∧^	577 (64.0)	262 (45.4)	1.81 (1.00, 3.29)		26 (4.5)	0.21 (0.09, 0.46)	
Well/stream/rain	118(13.1)	47 (39.8)	1.44 (0.73, 2.85)		5 (4.3)	0.20 (0.06, 0.61)	
River	153 (17.0)	66 (43.1)	1.65 (0.86, 3.19)		3 (2.0)	0.09 (0.02, 0.33)	
*Animal spend the night inside*	573 (63.2)	250 (43.6)	1.02 (0.78,1.34)	0.880	22 (3.9)	0.57 (0.31,1.04)	0.068
*Cooking inside the main house*	729 (80.8)	334 (45.8)	1.68 (1.18,2.37)	0.003	29 (4.0)	0.43(0.23,0.83)	0.012
*Soap use*				0.008			0.299
Daily	321 (35.4)	118 (36.8)	1	0.003^♀^	19 (5.9)	1	0.576^♀^
At least once a wk	532 (58.6)	249 (46.8)	1.51 (1.14, 2.01)		21 (4.0)	0.65 (0.35, 1.23)	
Less frequent than once a week	55 (6.1)	28 (50.9)	1.78 (1.00, 3.17)		4 (7.4)	1.27 (0.41, 3.88)	
*Household waste disposal(N = 904)*				0.013			0.996
Buried/Burned	190 (21.0)	65 (34.2)	1		9 (4.8)	1	
Open field	248 (27.3)	115 (46.4)	1.66 (1.23,2.46)		12 (4.8)	1.02 (0.42,2.47)	
Used as fertilizers	469 (51.7)	215 (45.8)	1.63 (1.15,2.31)		23 (4.9)	1.04 (0.47,2.28)	
*Maternal helminth infection*	395 (43.5)	-	-	-	22 (5.6)	1.32 (0.72,2.42)	0.368

^§^pit latrine, flush type and improvised pit latrine, ^♀^p for trend

^$^Inside pipe users are those who have a pipe inside the compound and usually used privately.

^∧^Outside pipe users are those whose source of water is from a pipe outside the compound mainly shared.

N = total number of study participants, n = number of outcome for each exposure

### Univariate risk factor analysis for STH infection in children

Significant univariate associations were identified with cooking site (OR 0.43; 95% CI 0.23-0.83 for inside relative to outside, p = 0.012), living in a house with a thatched roof (OR 0.51; 95% CI 0.27-0.96, p = 0.037) and with household source of water (overall p < 0.001) such that the greatest prevalence of infection was amongst those getting their water from a pipe inside the housing compound (18.5%), and the lowest in those using a river (3.9%) (Table 3). Being an urban resident was associated with increased risk of infection in the child (OR 2.07; 95% CI 1.00-4.32), but this was of borderline statistical significance (p = 0.052).

### Risk factors independently associated with STH in mothers

The independent predictors of maternal infection identified from multivariate analysis are shown in Table 4. Soap use (adjusted OR = 1.40; 95% CI 1.04-1.88 for infrequent users compared with daily users, p for trend = 0.018) and urban living (adjusted OR = 0.45; 95% CI 0.28-0.73, p = 0.001), remained significant factors.

**Table 4:** Multivariate analysis of independent risk factors for any soil-transmitted helminth infection in mothers and their children in the Butajira birth cohort, Ethiopia, 2007

Outcome variable	Risk factor	Adjusted OR	95% CI	P-value
Maternal infection	Soap use			0.055
	Daily	1		Trend = 0.018
	At least once a week	1.40	1.04, 1.88	
	Less frequent than once a week	1.66	0.92, 2.99	
	Urban residence*	0.45	0.28, 0.73	0.001
	Domestic animals living together	0.73	0.53, 1.01	0.055
	Age of the mother*			0.964
	15-24	1		Trend = 0.789
	25-34	0.97	0.72, 1.29	
	35-44	0.95	0.63, 1.44	
Child infection	Water source			0.002
	Inside pipe	1		
	Outside pipe	0.21	0.09, 0.51	
	Well/stream/rainwater	0.20	0.56, 0.69	
	River	0.09	0.02, 0.38	
	Urban residence*	1.03	0.43, 2.45	0.954
	Male gender*	0.67	0.36, 1.26	0.216

* Place of residence included as a priori confounder, and additionally age of the mother in the mother and gender in the child model

### Risk factors independently associated with STH in children

In multivariate analysis, after controlling for urban/rural residence and gender, the only significant predictor of infection in children was household source of water (p = 0.001) with the greatest risk of infection seen for those using piped water inside the compound (Table 4).

## Discussion

This study is the first, to our knowledge, to explore the prevalence of and risk factors for STH infection in an infant population in Ethiopia. The study found a high prevalence of hookworm infection among mothers, which exceeded the national average. However, the prevalence was generally low in children. The study indentified key environmental factors linked with STH infection which are amenable for intervention. Frequency of soap use and urban living were found to be predictors of maternal helminth infection while availability of piped water in the compound was independently associated with increased risk of infant helminth infection.

The findings of this study provide some evidence for environmental and public health measures to counter STH infection; yet they must be interpreted in the light of certain limitations. We lacked the power to identify risks weakly associated with infant infection in this cohort. Moreover, information on potential confounders was based on mothers' self-report, which may have led to information bias. Quantitative egg estimation to measure the intensity of infections using Kato-Katz technique was not performed due to issues related to feasibility. However, the results are unique in that they arise from a population based cohort, and provide valuable information on prevalence and risk factors among infants in a low-income setting.

The prevalence of STH infection observed in mothers (43.5%) was higher than that reported previously (33.8%) in a random sample of the Butajira Rural Health Project [18]. In this previous study, hookworms were found in 14.7% of the study population, lower than in our mothers (36.1%) while *A. lumbricoides *was found in a higher proportion of the general population (16.6%) than in our mothers (8.8%). The low prevalence of hookworm in the previous study may be because the earlier study population was on average younger than the mothers in this study (median age 20 vs. 26 years). Moreover, the previous study consisted of a significantly higher proportion of urban participants, who live in houses with corrugated iron roofs, and were more educated than the birth cohort women. All these factors might explain the lower prevalence of hookworm infection in the earlier study [18].

Multiple STH infections were also less common than in other studies in Ethiopia. Studies in the south and areas around Wondo-Genet report 80% of populations to be infected with one helminthic parasite, 40% with two, and 3% with four [19,20]. Such differences might arise from differences in study subjects, socio-demographic conditions of the society or differences in the parasitological examination techniques.

The prevalences of hookworm (2.3%) and *A. lumbricoides *(1.5%) in our infants were much lower than those found among one year-old children in Jimma, Ethiopia, (hookworm 13.3%, *A. lumbricoides *35.0%, [21]). The lower prevalence of STHs in children in this population is probably linked to the Enhanced Outreach Strategy (EOS) for child survival, a mass de-worming intervention started in 2006 in under-five children in this area [5]. Even though we were not clearly able to ascertain whether the deworming included our study participants, the intervention might have changed the parental health related behavior or siblings in the household might have received the medication further reducing transmission.

This study showed that maternal hookworm infection was more prevalent (36.1%) than other STH infection. In their review, Hotez *et al *indicated that 30% of Kenyan, 41% of Nepalese, and 53% of Vietnamese women of reproductive age had a hookworm infection [2], higher than other STHs and comparable to this study. This may be explained by the fact that women spend more time than men in muddy, wet gardens suitable for third stage infective hookworm larvae. Women in the Butajira area spend much of their time in farm plots and wet gardens covered by false banana, *Khat *and coffee trees that may all contribute to the survival of hookworm larvae. The higher prevalence of hookworm infection in adulthood may also be explained by the nature of the parasite-host relationship [2]: unlike other soil-transmitted helminths, hookworms secrete many bioactive polypeptides which dampen down host immune responses [2].

The present study also assessed the possible association of STH infection with potential risk factors among mothers and children in the cohort. Several recent studies have identified a range of environmental and social risk factors associated with STH infections [22-24]. However, very few of the environmental and socioeconomic factors were significantly associated in this study; making it comparable to earlier studies in Ethiopia and other less developed countries [23,25,26]. One of the factors strongly protecting against maternal helminth infection in this study was daily use of soap by the mothers. It was not the presence of soap in the household but the frequency of its use that protected from STH infection. Several studies have shown a significantly higher prevalence of helminth infection among subjects who rarely washed their hands with soap compared to those who washed their hands regularly [22,25-27].

The other factor protecting mothers from helminth infection was urban residence. This is likely to be related to the lower prevalence of sanitary measures in the rural areas of Ethiopia. For instance, the proportion of the population with access to an improved water source is nearly twice as high in urban as in rural areas [13]. In addition, access to improved sanitation is four times higher in urban than rural areas [13]. Interventions to reduce helminth infection must focus on rural residents. This study also found that using water from a pipe inside a compound was a risk factor for helminth infection in infants. Other studies have shown that helminth infection among pipe users in underdeveloped countries may arise from the poor quality of piped water [28,29]. Efforts to minimize microbial contamination of piped water supplies and to monitor water quality are important. However, other unknown factors may contribute to the increased risk associated with piped water and merit further investigation.

## Conclusions

STH infection is common in mothers in this low-income birth cohort. Risk factors associated with infection suggest that mass de-worming strategies must also address provision of safe water and health education about soap use, particularly among rural residents.

## Competing interests

The authors declare that they have no competing interests.

## Authors' contributions

GD, JB, AV, CH & GM conceived the idea for this study. GM, GD, CH, BE, YB and AA participated in the design and conduct of the study. YB and GM were responsible for the accuracy of the data. AAm and YB drafted the manuscript. AAm and AV guarantee the statistical analysis. AAm, GD, AV, JB, YB and BE interpreted the findings. All authors read and approved the final manuscript.

## Pre-publication history

The pre-publication history for this paper can be accessed here:

http://www.biomedcentral.com/1471-2458/10/21/prepub
